# Rationale, design, and baseline characteristics of a randomized, placebo-controlled cardiovascular outcome trial of empagliflozin (EMPA-REG OUTCOME™)

**DOI:** 10.1186/1475-2840-13-102

**Published:** 2014-06-19

**Authors:** Bernard Zinman, Silvio E Inzucchi, John M Lachin, Christoph Wanner, Roberto Ferrari, David Fitchett, Erich Bluhmki, Stefan Hantel, Joan Kempthorne-Rawson, Jennifer Newman, Odd Erik Johansen, Hans-Juergen Woerle, Uli C Broedl

**Affiliations:** 1 Lunenfeld-Tanenbaum Research Institute, Mount Sinai Hospital, Toronto, Canada; 2 Division of Endocrinology, University of Toronto, Toronto, Canada; 3 Section of Endocrinology, Yale University School of Medicine, New Haven, CT, USA; 4 The Biostatistics Center, The George Washington University, Rockville, MD, USA; 5 Department of Medicine, Division of Nephrology, Würzburg University Clinic, Würzburg, Germany; 6 Section of Cardiology, University of Ferrara, Ferrara, Italy; 7 St Michael’s Hospital, Toronto, Canada; 8 Division of Cardiology, University of Toronto, Toronto, Canada; 9 Boehringer Ingelheim Pharma GmbH & Co. KG, Biberach, Germany; 10 Boehringer Ingelheim Pharmaceuticals, Inc, Ridgefield, CT, USA; 11 Boehringer Ingelheim Norway KS, Asker, Norway; 12 Boehringer Ingelheim Pharma GmbH & Co. KG, Ingelheim, Germany

**Keywords:** Blood pressure, Body weight, Empagliflozin, Glycemic control, Macrovascular, Microvascular, SGLT2 inhibitor, Type 2 diabetes

## Abstract

**Background:**

Evidence concerning the importance of glucose lowering in the prevention of cardiovascular (CV) outcomes remains controversial. Given the multi-faceted pathogenesis of atherosclerosis in diabetes, it is likely that any intervention to mitigate this risk must address CV risk factors beyond glycemia alone. The SGLT-2 inhibitor empagliflozin improves glucose control, body weight and blood pressure when used as monotherapy or add-on to other antihyperglycemic agents in patients with type 2 diabetes. The aim of the ongoing EMPA-REG OUTCOME™ trial is to determine the long-term CV safety of empagliflozin, as well as investigating potential benefits on macro-/microvascular outcomes.

**Methods:**

Patients who were drug-naïve (HbA_1c_ ≥7.0% and ≤9.0%), or on background glucose-lowering therapy (HbA_1c_ ≥7.0% and ≤10.0%), and were at high risk of CV events, were randomized (1:1:1) and treated with empagliflozin 10 mg, empagliflozin 25 mg, or placebo (double blind, double dummy) superimposed upon the standard of care. The primary outcome is time to first occurrence of CV death, non-fatal myocardial infarction, or non-fatal stroke. CV events will be prospectively adjudicated by an independent Clinical Events Committee. The trial will continue until ≥691 confirmed primary outcome events have occurred, providing a power of 90% to yield an upper limit of the adjusted 95% CI for a hazard ratio of <1.3 with a one-sided α of 0.025, assuming equal risks between placebo and empagliflozin (both doses pooled). Hierarchical testing for superiority will follow for the primary outcome and key secondary outcomes (time to first occurrence of CV death, non-fatal myocardial infarction, non-fatal stroke or hospitalization for unstable angina pectoris) where non-inferiority is achieved.

**Results:**

Between Sept 2010 and April 2013, 592 clinical sites randomized and treated 7034 patients (41% from Europe, 20% from North America, and 19% from Asia). At baseline, the mean age was 63 ± 9 years, BMI 30.6 ± 5.3 kg/m^2^, HbA1c 8.1 ± 0.8%, and eGFR 74 ± 21 ml/min/1.73 m^2^. The study is expected to report in 2015.

**Discussion:**

EMPA-REG OUTCOME™ will determine the CV safety of empagliflozin in a cohort of patients with type 2 diabetes and high CV risk, with the potential to show cardioprotection.

**Trial registration:**

Clinicaltrials.gov NCT01131676

## Introduction

Type 2 diabetes mellitus (T2DM) is frequently associated with comorbidities that exacerbate cardiovascular (CV) risk, such as obesity and hypertension [1]. The risk of CV disease is increased approximately two to four-fold in adults with diabetes even after adjustment for conventional risk factors (age, sex, smoking status, body mass index [BMI], systolic blood pressure [BP], and lipids) [2]. Recommended strategies for reducing CV risk in patients with T2DM include glucose management, lipid lowering, BP control, smoking cessation, and weight loss [1]. Improved glycemic control has been associated with a reduction in microvascular events [3] and there is a clear association between microvascular complications such as albuminuria and an increased risk of CV events in patients with T2DM [4]. However, the impact of reducing blood glucose, and the potential benefit of specific glucose-lowering agents, on CV events in patients with T2DM remains unclear and highly controversial [5,6]. Moreover, treatment must likely occur over a substantial duration of time, since macrovascular outcome events are known to be late complications of a progressive multifaceted pathogenic process that spans decades [7,8]. Lately, regulatory authorities have issued guidance for evaluating the long-term CV safety of new anti-diabetes agents to ensure that CV safety is demonstrated with reasonable assurance [9,10]. These mandated trials provide an opportunity to potentially demonstrate CV as well as microvascular benefits of new anti-diabetes drugs.

Sodium glucose cotransporter 2 (SGLT2) inhibitors are a new class of antidiabetes agents that reduce hyperglycemia in patients with T2DM by reducing renal glucose reabsorption and thus increasing urinary glucose excretion (UGE) [11]. Empagliflozin is a potent and selective inhibitor of SGLT2 [12]. In placebo-controlled phase III trials in patients with T2DM, empagliflozin used as monotherapy or add-on therapy improved hemoglobin A1c (HbA1c) approximately 0.7-1.0% -point (depending on baseline HbA1c and renal function) with a low risk of hypoglycemia, reduced body weight and BP, without increases in heart rate, and had small effects on plasma lipids (increase in HDL-cholesterol, increase in LDL-cholesterol, no change in LDL/HDL cholesterol ratio) [13-17]. In addition, empagliflozin has been shown to improve arterial stiffness and reduce glomerular hyperfiltration in patients with type 1 diabetes mellitus (T1DM) [18,19]. Moreover, SGLT2 inhibitors have also been reported to reduce other CV risk markers such as visceral fat mass [20,21] and proteinuria [22]. Based on these pleiotropic effects on CV risk factors, we hypothesized that empagliflozin may reduce CV risk in patients with T2DM.

The EMPA-REG OUTCOME™ trial was designed to determine the long-term CV safety of empagliflozin in patients with T2DM and to investigate its potential cardioprotective effects, as well as impact on microvascular outcomes, in a dedicated study that complied with current regulatory requirements.

## Methods

The EMPA-REG OUTCOME™ trial (clinicaltrials.gov identifier: NCT01131676) is an ongoing, multicenter, randomized, double-blind, placebo-controlled trial. It was designed to assess the effect of empagliflozin (10 mg or 25 mg once daily) compared with placebo, in addition to standard of care, on CV events in adults with T2DM at high risk of CV events and with less than optimized glycemic control.

The study protocol was approved by the respective Institutional Review Boards, Independent Ethics Committees and Competent Authorities according to national and international regulations.

### Trial population

Our goal was to recruit 7000 participants across 42 countries. Patients aged ≥18 years (≥20 years in Japan and also ≤65 years in India) with T2DM who were drug-naïve (no anti-diabetes agents for ≥12 weeks prior to randomization) with HbA1c ≥7.0% and ≤9.0% or taking any background anti-diabetes therapy (except pioglitazone in Japan) with HbA1c ≥7.0% and ≤10.0% despite diet and exercise counseling and who were at high risk of CV events were eligible for inclusion. The main inclusion criteria are provided in detail in Table 1. The dose of background glucose-lowering therapy was required to be unchanged for ≥12 weeks prior to randomization or, in the case of insulin, unchanged by >10% from the dose at randomization in the previous 12 weeks. Subjects were required to have a BMI ≤45 kg/m^2^ at baseline. Detailed inclusion and exclusion criteria are listed in Additional file 1.

**Table 1:** Key inclusion criteria

**Insufficient glycemic control**	**High risk of cardiovascular events (≥1 of the following)**
• ***Drug-naive subjects*:** HbA_1c_ ≥7.0% and ≤9.0% at screening	• History of myocardial infarction >2 months prior to informed consent
	• Evidence of multi-vessel CAD i.e. in ≥ 2 major coronary arteries or the left main coronary artery, documented by any of the following:
• ***Subjects on background therapy*:** HbA_1c_ ≥7.0% and ≤10.0% at screening	– Presence of significant stenosis: ≥50% luminal narrowing during angiography (coronary or multi-slice computed tomography)
	– Previous revascularization (percutaneous transluminal coronary angioplasty ± stent or coronary artery bypass graft >2 months prior to consent
	– The combination of revascularization in one major coronary artery and significant stenosis (≥50% luminal narrowing) in another major coronary artery
	• Evidence of single-vessel CAD, ≥50% luminal narrowing during angiography (coronary or multi-slice computed tomography) not subsequently successfully revascularized, with at least 1 of the following:
	– A positive non-invasive stress test for ischemia
	– Hospital discharge for unstable angina ≤12 months prior to consent
	• Unstable angina >2 months prior to consent with evidence of single- or multi-vessel CAD
	• History of stroke (ischemic or hemorrhagic) >2 months prior to consent
	• Occlusive peripheral artery disease documented by any of the following:
	– Limb angioplasty, stenting, or bypass surgery
	– Limb or foot amputation due to circulatory insufficiency
	– Evidence of significant peripheral artery stenosis (>50% on angiography, or >50% or hemodynamically significant via non-invasive methods ) in 1 limb
	– Ankle brachial index <0.9 in ≥1 ankle

CAD, coronary artery disease.

### Study design

**Figure 1:**
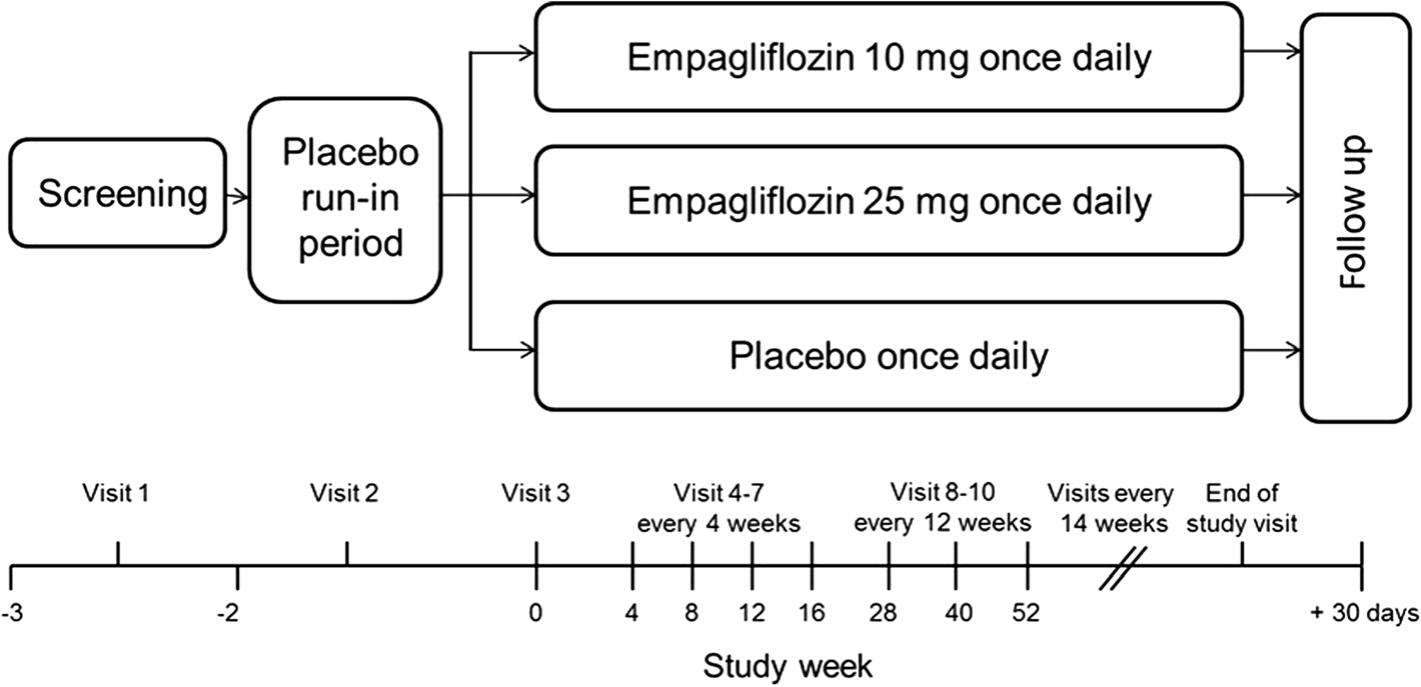
Study design.

Eligible patients underwent a 2-week, open-label, placebo run-in period (Figure 1) during which background glucose-lowering therapy was continued unchanged. The purpose of the run-in period was to evaluate participants’ willingness to adhere to the long-term treatment and follow-up planned in the trial. Following the placebo run-in, patients still meeting the inclusion/exclusion criteria were randomized (1:1:1) to receive empagliflozin 10 mg, empagliflozin 25 mg, or placebo once daily in addition to their background therapy. Background glucose-lowering therapy was to remain unchanged for the first 12 weeks after randomization if possible, although rescue therapy could be initiated (details in Additional file 2). After this period, therapy could be adjusted to achieve desired glycemic control at the investigator’s discretion to achieve best standard of care according to local guidelines. Investigators were encouraged to treat all other CV risk factors according to local standard of care.

### Randomization and follow-up

Randomization was undertaken using a computer-generated random sequence and an interactive voice and web response system. Patients were stratified by HbA_1c_ at screening (<8.5%, ≥8.5%), BMI at randomization (<30 kg/m^2^, ≥30 kg/m^2^), region (North America [plus Australia and New Zealand], Latin America, Europe, Africa, Asia), and renal function (eGFR using the Modification of Diet in Renal Disease [MDRD] equation) at screening (Chronic Kidney Disease [CKD] stage 1: ≥90 ml/min/1.73 m^2^; CKD stage 2: 60–89 ml/min/1.73 m^2^; CKD stage 3: 30–59 ml/min/1.73 m^2^). Patients are instructed to attend the clinic at pre-specified times over the duration of the study including a follow-up visit 30 days after the end of the treatment period (Figure 1). Patients who prematurely discontinue study medication are asked to attend all visits as originally planned.

### Outcomes and outcome adjudication

The primary outcome of the study is time to first occurrence of CV death, non-fatal myocardial infarction (MI, excluding silent MI), or non-fatal stroke *i.e.,* 3-point major adverse cardiovascular events (3P-MACE). The key secondary outcome expands the primary composite outcome to include time to first occurrence of hospitalization for unstable angina (4P-MACE). Further CV outcomes are the individual components of the 4P-MACE, as well as individual occurrence of and time to silent MI, heart failure requiring hospitalization, all-cause mortality, transient ischemic attack (TIA) and coronary revascularization procedures. All CV outcome events and deaths are being prospectively adjudicated by the Clinical Events Committee (one for cardiac events and one for neurological events), as recommended in FDA guidelines (FDA [9]). Definitions of the major clinical outcomes are presented in Additional file 3 and a non-exhaustive list of further CV outcomes (secondary, tertiary and exploratory) in Additional file 4.

Additional secondary outcomes include the occurrence of and time to new onset albuminuria (urinary albumin:creatinine ratio ≥30 mg/g) and new onset of macroalbuminuria (urinary albumin:creatinine ratio ≥300 mg/g). Other outcomes include the occurrence of and time to a composite microvascular outcome comprising the initiation of laser therapy for retinopathy, vitreous hemorrhage, diabetes-related blindness, and new or worsening nephropathy (new onset macroalbuminuria [albumin:creatinine ratio ≥300 mg/g]; doubling of serum creatinine accompanied by eGFR ≤45 mL/min/1.73 m^2^; initiation of renal replacement therapy; or death due to renal disease) as well as the individual components of this composite.

The short (12 weeks), medium (52 weeks), and long-term (annually, at end of study, and at follow-up) effects of the two doses of empagliflozin on HbA_1c_, fasting plasma glucose (FPG), body weight, waist circumference, and BP will be assessed, as well as the proportion of patients who meet the composite outcome of HbA_1c_ reduction ≥0.5%, systolic BP reduction >3 mmHg, and body weight reduction >2%.

The prognostic impact of, and the modulating potential of empagliflozin on, the renal biomarker cystatin C and the CV biomarkers high-sensitivity C-reactive protein and high sensitivity troponin T will be assessed in sub-studies, as will potential associations between genetic variations and drug response.

Safety will be assessed based on adverse events (AEs) reported throughout the study and up to 7 days after the last dose of study medication (coded using the Medical Dictionary for Drug Regulatory Activities [MedDRA]), clinical laboratory tests, vital signs, 12-lead electrocardiogram (ECG), physical examination, and the use of rescue medication. Pre-specified AEs of special interest (AESI) include confirmed hypoglycemic adverse events (plasma glucose ≤70 mg/dL (3.9 mmol/L) and/or requiring assistance), those reflecting volume depletion, bone fracture, hepatic events, malignancies, urinary tract infection (UTI), and genital infection. Events may be defined by either abnormal laboratory values and/or relevant adverse events identified using prospectively defined search categories or both. For qualifying events, relevant source documentation will be requested including lab values, histological analysis, results of ultrasound, CT, MRI, scintigraphy, hospital discharge letters, and medical reports from other physicians. All evaluations will be performed in a blinded fashion.

A list of efficacy and safety outcomes is presented in Additional file 4.

### Study oversight and organization

The trial was jointly designed by employees of Boehringer Ingelheim (BI) and the academic investigators who were members of the Steering Committee. The Steering Committee, which was led by the academic investigators and included members who were employees of the sponsor, supervised the trial design and operation. The independent data and safety monitoring committee (DMC) reviews interim safety data every 90 days or on an ad hoc basis on request. A list of committees involved in the trial conduct is presented in Additional file 5.

### Statistical considerations

#### Sample size and power calculations

The primary hypothesis aims to show non-inferiority on 3P-MACE for empagliflozin versus placebo based on a non-inferiority margin of < 1.3 (upper limit of the adjusted 95% confidence interval (CI)) for the hazard ratio. The upper limit of the adjusted 95% CI for the HR of <1.3 was based on FDA guidance for CV trials evaluating new anti-hyperglycemic therapies for T2DM [9]. Patients who receive either 10 mg or 25 mg of empagliflozin will be pooled into a common treatment group for the purposes of the primary analysis. A 4-step hierarchical testing strategy will be followed: 1) non-inferiority test of the primary outcome (3P-MACE), 2) non-inferiority test of the key secondary outcome (4P-MACE), 3) superiority test of the primary outcome (3P-MACE) and 4) superiority test of the key secondary outcome (4P-MACE). A minimum of 691 confirmed primary outcome events are required to provide 90% power with a one-sided α level of 0.025, assuming equal risk between the placebo and empagliflozin groups. With a minimum of 691 events, the trial will also have at least 80% power to detect a hazard ratio of 0.785 (corresponding to a 21.5% risk reduction in CV outcome events) for the primary outcome.

#### Interim analysis

In order to support a CV meta-analysis of all CV events occurring in the phase III trials involving empagliflozin, as required for all New Drug Applications to be submitted to the FDA [9], CV outcome data from the ongoing EMPA-REG OUTCOME™ was extracted. The cut off for the data extraction was preplanned and ~ 150 4P-MACE were included in the project level CV meta-analysis. This resulted in addition of a Haybittle-Peto correction for the interim analysis (i.e., 0.0001 of the α was spent on the data extraction for the interim analysis), and subsequent reduction of the final α level to 0.0249 (in order to maintain the experiment-wise α level of 0.025).

The need to prevent the release of any data from the data extraction or interim analysis that could define the effects of empagliflozin on CV outcomes was fundamental to the study design [23]. Accordingly, procedures, including restricted access to electronic systems, were put in place to ensure that the effect estimate remained blinded and data review by the regulators would not require premature disclosure of the effects of empagliflozin on CV outcome. The data extraction, interim analysis and the following phase III CV meta-analysis were performed by a group independent from the EMPA-REG OUTCOME™ trial team, so that the trial’s operational team and the academic Steering Committee remained blinded to the results. The DMC is the sole group with access to unblinded results beyond the strictly firewalled “CV meta-analysis group” of the sponsor.

#### Analysis plan

Three analysis populations are defined for this trial: 1) The treated set (TS), consisting of all patients who were treated with at least one dose of study drug, 2) The on-treatment set (OS), consisting of patients who received the drug for at least 30 days (cumulative) in whom events will be considered that occurred within 30 days of the off-treatment period or until the end of the entire trial, whichever will be earlier (patients who did not experience the primary outcome will be censored at the end of the treatment period, if the patient completes treatment as planned, or at the end of the 30 day period) and 3) the Full Analysis Set (FAS), consisting of all patients randomized, treated with at least one dose of study drug and with a baseline HbA1c value.

The primary analysis will be based on a Cox proportional hazards model with treatment (with empagliflozin 10 mg and 25 mg pooled into a single group), age, gender, baseline BMI (<30 kg/m^2^, ≥30 kg/m^2^), baseline HbA_1c_ (<8.5%, ≥8.5%), baseline eGFR as well as geographical region (classified as North America, Latin America, Europe, Africa and Asia) as factors. The same Cox proportional hazards model as for the primary outcome will be employed in all steps of the hierarchical testing strategy (3P-MACE and 4P-MACE).

The time to the occurrence of the primary outcome and the key secondary outcomes event will be computed as (event date - randomization date) +1. Patients who do not have the event during the trial period will be censored at the individual day of trial completion. The time to censoring will be computed as (individual day of trial completion – randomization date) + 1. For patients who have more than one primary outcome event during the trial, the time to the first occurrence of the primary outcome event will be considered for the primary analysis. All adjudicated and confirmed events will be used for the primary analysis.

The TS is the basis for the primary analysis and the FAS is the basis for the intention-to-treat (ITT) analysis for efficacy analyses. As sensitivity analyses the primary analysis will also be performed for the OS. Secondary analyses of the primary analysis with pooled active treatment arms will be performed by comparing the active treatments individually versus the placebo arm. Sensitivity analyses of the primary and key secondary outcomes will be performed that include the additional factor of naïve/experienced drug status in the Cox model. The secondary and tertiary further cardiovascular outcomes will be analyzed in a Cox proportional hazards model similar to the primary analysis for the treated set. Of note is that also other sensitivity analysis will be conducted employing other statistical methods, as well as an assessment of outcomes per individual dosages (i.e., empagliflozin 10 mg and empagliflozin 25 mg).

Subgroups to be considered in the analyses will be defined based on, but not limited to, age, HbA1c, BMI, weight, geographical region, race, gender, ethnicity, time since diagnosis of T2DM, renal function, BP, eGFR, glucose-lowering and CV prophylactic medication, CV complications and cohort, all defined at study baseline or screening. In addition, outcomes in patients experiencing severe hypoglycaemia vs those not experiencing severe hypoglycaemia will be assessed. Further details as to the specific categories to be employed for each subgroup factor are provided in Additional file 6.

### Patient recruitment and baseline characteristics

Recruitment into the EMPA-REG OUTCOME™ trial began in September 2010 and was completed in April 2013. In total 11507 patients were screened and 7042 participants were randomized to receive study treatment at 592 clinical sites in 42 countries. The main reason for screen-failure was that the HbA1c fell outside protocol specifications. Of those randomized, 7034 participants were treated. The baseline characteristics of treated participants are shown in Table 2. Most came from Europe (41%) or North-America (20%) with 19% from Asia, 15.4% from Latin America and 4% from South-Africa. The mean age of participants was 63 years, with 9% aged ≥75 years. Seventy-two per cent are male, and 72% are white. Time since diagnosis of T2DM was ≤5 years in 18% of participants and >10 years in 57%. At baseline, mean HbA_1c_ was 8.1% (Table 3) with 68% of participants having HbA_1c_ <8.5%. Only 2% of participants were drug-naïve; 29% were receiving monotherapy, and 45% were receiving dual therapy. Insulin was used by 36% of participants (as monotherapy or part of dual therapy). A history of CV complications or CV events was demonstrated in 99% of participants and in total 47% had a history of MI and 23% a history of stroke. Fifty-two per cent of participants had an eGFR ≥ 60 and <90 mL/min/1.73 m^2^ (i.e., stage 2 CKD) and 26% had an eGFR ≥ 30 and <60 mL/min/1.73 m^2^ (i.e., stage 3 CKD). Albuminuria (UACR ≥30 mg/g) was present in 40% of participants. At baseline, 77% of patients were receiving a statin, 9% were receiving a fibrate, 85% were being treated with an acetylsalicylic acid agent, and 94% were receiving any drug for BP reduction (80% on blockers of the renin-angiotensin system).

**Table 2:** Baseline characteristics (treated set; n = 7034)

Age (years), mean (SD)	63.1 (8.6)
≥ 75 years of age, n (%)	652 (9)
Male, n (%)	5026 (72)
Race, n (%)	
White	5089 (72)
Asian	1518 (22)
Black/African American	357 (5)
Other*	70 (1)
Ethnicity, n (%)	
Hispanic or Latino	1268 (18)
Smoking history, n (%) Current/Ex-smoker	930 (13)/3216 (46)
Time since diagnosis, n (%)	
≤5 years	1265 (18)
>5-10 years	1754 (25)
>10 years	4015 (57)
Region, n (%)	
Europe	2885 (41)
North America/Australia/New Zealand	1408 (20)
Latin America	1081 (15)
Africa	313 (4)
Asia	1347 (19)
Northeast Asia	586 (8)
South/South-East Asia	761 (11)
CV risk factors, any of the below, n (%)	6978 (99)
History of MI	3275 (47)
Single-vessel CAD	743 (11)
Multi-vessel CAD	3285(47)
CABG	1738(25)
History of stroke	1631 (23)
Peripheral occlusive arterial disease	1449 (21)
Glucose-lowering therapy at baseline, n (%)	
None	128 (2)
Monotherapy	2055 (29)
Metformin (% of monotherapy)	745 (36)
Insulin (% of monotherapy)	954 (46)
Dual therapy	3188 (45)
Metformin + sulfonylurea (% of dual therapy)	1383 (43)
Metformin + insulin (% of dual therapy)	1420 (45)
Other therapies (n, %)	
Acetylsalicylic acid	5990 (85)
Statins	5387 (77)
Fibrates	630 (9)
Any antihypertensive therapy (n, %)	6641 (94)
Blockers of the renin-angiotensin system	5651 (80)
Beta-blockers	4537 (64)
Calcium channel blockers	2114 (30)

*American Indian/Native Alaskan/Native Hawaiian/Pacific Islander/missing.

Results (based on a pre-final version of the database of this ongoing trial) may change slightly once trial is completed.

**Table 3:** Key baseline laboratory data (treated set; n = 7034)

HbA_1c _(%), mean (SD)	8.1 (0.8)
HbA_1c_ <8.5%, n (%)	4811 (68)
Fasting plasma glucose (mmol/L), mean (SD)	8.5 (2.4)
Body mass index (kg/m^2^), mean (SD)	30.6 (5.3)
≥ 35 kg/m^2^, n (%)	1426 (20)
Weight (kg), mean (SD)	86.4 (18.9)
Waist circumference (cm), mean (SD)	105 (14)
Systolic/diastolic blood pressure (mmHg), mean (SD)	135 (17)/77 (10)
Lipids (mmol/L), mean (SD)	
Total cholesterol	4.2 (1.1)
LDL-cholesterol	2.2 (0.9)
HDL-cholesterol	1.2 (0.3)
Triglycerides	1.9 (1.4)
eGFR according to MDRD (mL/min/1.73 m^2^), mean (SD)	74 (21)
eGFR according to MDRD (mL/min/1.73 m^2^), n (%)	
≥90	1534 (22)
60 to <90	3671 (52)
30 to <60	1796 (26)
ACR albumin ratio (mg/g), median (Q1, Q3)	17.7 (7.1, 72.5)
ACR ratio (mg/g), n (%)	
≥ 30 – 300	2011 (29)
≥ 300	771 (11)

Results (based on a pre-final version of the database of this ongoing trial) may change slightly once trial is completed.

## Discussion

The EMPA-REG OUTCOME™ trial is an ongoing, randomized, placebo-controlled, clinical outcomes trial powered to establish the CV safety of empagliflozin with the potential to demonstrate cardioprotection in patients with T2DM at high risk of CV events who are receiving standard of care.

The pragmatic inclusion of patients on any background glucose-lowering agents will enable an assessment of the long-term CV effects of empagliflozin in a representative cohort and in a setting similar to real-life clinical practice. Of further note is that the trial will be able to assess the impact of empagliflozin on CV risk, in particularly vulnerable patient groups since ~ 25% patients have eGFR < 60 and ~10% were ≥75 years of age at baseline. Further, given the diversity of background therapy being allowed, CV outcomes according to type of background therapy can be derived. Recruitment into the study is complete and the baseline characteristics of the 7034 treated participants indicate that, as planned, they are at high risk of CV events and we anticipate that the pre-specified number of 3P-MACE will be reached in 2015. Thus, this trial will be one of the first, if not the first, to report final CV outcome data amongst the ongoing SGLT2i CV outcome trials: DECLARE-TIMI58 (clinicaltrials.gov identifier: NCT01730534) involving dapagliflozin, CANVAS (clinicaltrials.gov identifier: NCT01032629) involving canagliflozin [24] and the ertugliflozin CV outcome study (clinicaltrials.gov identifier: NCT01986881), which all according to public sources will complete 2017–2020.

With 7034 patients enrolled and treated, the trial is in keeping with the 2008 FDA guidance on evaluating the CV risk of new therapies to treat T2DM [9] but may also provide insights beyond CV safety, including impact on microvascular, in particular renal outcomes, as detailed above. A theoretical basis for renal protection with SGLT2 inhibitors has been proposed, encompassing reduction in tubular stress as well as glucose-induced inflammation and fibrotic markers in the proximal tubule *in vitro* and in animal models, as well as improvement in glucose and BP control, reduction in plasma uric acid and albuminuria, and reduction in glomerular hyperfiltration with improvement in glomerular capillary hypertension [19,22,25-28].

Since the majority (i.e., 78%) of participants in EMPA-REG OUTCOME™ had some degree (i.e. CKD 2 or 3) of renal impairment at baseline, including 11% with macroalbuminuria, this trial is also expected to provide valuable information on the effect of empagliflozin on renal outcomes. Of note, renal outcomes comprise the dedicated scope for two other SGLT2i outcome trials, i.e., the CANVAS-R trial (clinicaltrials.gov identifier NCT01989754) which will investigate the effects of canagliflozin on progression of albuminuria in 5700 patients with T2DM and the CREDENCE trial (clinical trials.gov identifier NCT02065791) which will investigate the effects of canagliflozin on the incidence of end-stage kidney disease, serum- creatinine doubling and renal and CV death in 3627 patients with T2DM and stage 2 and 3 CKD and macroalbuminuria, estimated to report in 2017 and 2019, respectively.

In summary, it is expected that the results of the EMPA-REG OUTCOME™ trial will provide evidence concerning the CV safety of empagliflozin, as well as provide insights on the potential benefits of empagliflozin on CV and microvascular outcomes. Thus the results of the EMPA-REG OUTCOME™ trial will help to inform clinical decision-making for patients with T2DM.

## Abbreviations

3P-MACE: 3-point major adverse cardiovascular events; 4P-MACE: 4-point major adverse cardiovascular events; ACR: Albumin/creatinine ratio; AE: Adverse event; AESI: Adverse events of special interest; BI: Boehringer Ingelheim; BP: Blood pressure; BMI: Body mass index; CV: Cardiovascular; CG: Cockcroft-Gault; CKD: Chronic kidney disease; DBP: Diastolic blood pressure; DMC: Data monitoring committee; eGFR: Estimated glomerular filtration rate; FAS: Full analysis set; FPG: Fasting plasma glucose; HbA1c: Glycosylated hemoglobin; HR: Hazard ratio; ITT: Intention to treat; LOCF: Last observation carried forward; MACE: Major adverse cardiovascular events; MDRD: Modified diet renal disease formula; MI: Myocardial infarction; MMRM: Mixed model repeated measures; OS: On-treatment set; qd: Once daily; SBP: Systolic blood pressure; SGLT2: Sodium glucose cotransporter 2; SGLT2i: Sodium glucose cotransporter 2 inhibitor; T2DM: Type 2 diabetes mellitus.

## Competing interests

BZ, SEI, JML, CW, RF and DF have received fees for advisory services to BI. EB, SH, JKH, JN, OEJ, HJW and UCB are employees of BI, the developer of empagliflozin.

## Authors’ contributions

All authors contributed to the development of the manuscript and read and approved the final manuscript.

## Supplementary Material

Additional file 1 Inclusion and exclusion criteria. Click here for file

Additional file 2 Criteria for the institution of rescue therapy. Click here for file

Additional file 3 Outcome definitions for major clinical outcomes. Click here for file

Additional file 4 Study outcomes (non-exhaustive list). Click here for file

Additional file 5 Study organization. Click here for file

Additional file 6 Selected subgroups of interest. Click here for file
