# Supplementary material for: Rationale, design, and baseline characteristics of a randomized, placebo-controlled cardiovascular outcome trial of empagliflozin (EMPA-REG OUTCOME™)
Source: Cardiovasc Diabetol. 2014 Jun 19;13:102. doi: 10.1186/1475-2840-13-102 (PMC4072621; doi:10.1186/1475-2840-13-102)
Supplement: Additional file 1 — Inclusion and exclusion criteria. [file 1475-2840-13-102-S1.docx]

**Additional file 1. Inclusion and exclusion criteria**

**Inclusion criteria**

All of the following criteria needed to be met:

- Subjects with T2DM on a diet and exercise regimen who are drug-naïve (no anti-diabetes therapy for ≥12 weeks prior to randomization) or treated with any anti-diabetes therapy (except pioglitazone in Japan)
  - Drug-naive patients were required to have HbA_1c_ ≥7.0% (≥53 mmol/mol) and ≤9.0% (≤75 mmol/mol) at screening
  - Pre-treated patients were required to have HbA_1c_ ≥7.0% (≥53 mmol/mol) and ≤10.0 (≤86 mmol/mol) at screening
    - Background anti-diabetes therapy had to be unchanged for ≥12 weeks prior to randomization
    - If background anti-diabetes therapy included insulin, insulin dose had to be unchanged by >10% from the dose at randomization in the previous 12 weeks
- Age ≥18 years (≥20 years in Japan and ≤65 years in India)
- Body mass index ≤45 kg/m^2^ at screening
- High risk of a cardiovascular event defined as ≥1 of the following:
  - Confirmed history of myocardial infarction (>2 months prior to informed consent)
  - Evidence of multivessel coronary artery disease (CAD), in ≥2 major coronary arteries or in the left main coronary artery, irrespective of revascularization status, i.e. one of the following:
    - Presence of a significant stenosis (imaging evidence of ≥50% narrowing of the luminal diameter measured during a coronary angiography or a multi-sliced computed tomography [CT] angiography)
    - Revascularization (percutaneous transluminal coronary angioplasty [PTCA] with or without stent, or coronary artery bypass grafting [CAGB]) ≥2 months prior to consent
    - Combination of revascularization in one major coronary artery ≥2 months prior to consent (PCTA with or without stent, or CABG), and the presence of significant stenosis in another major coronary artery (imaging evidence of ≥50% narrowing of the luminal diameter measured during a coronary angiography or a multi-sliced CT angiography)
  - Evidence of a single vessel CAD with the presence of significant stenosis i.e. imaging evidence of ≥50% narrowing of the luminal diameter of one major coronary artery in patients not subsequently successfully revascularized (measured during a coronary angiography or a multi-sliced CT angiography) and one or both of the following:
    - A positive non-invasive stress test, confirmed by one of the following:
      - Positive exercise tolerance test in subjects without a complete left bundle branch block, Wolff-Parkinson-White syndrome, or paced ventricular rhythm
      - Positive stress echocardiography showing regional systolic wall motion abnormalities
      - Positive scintigraphic test showing stress-induced ischemia, i.e. development of transient perfusion defects during myocardial perfusion imaging
    - Patient discharged from hospital with a documented diagnosis of unstable angina within 12 months of consent
  - Last episode of unstable angina >2 months prior informed consent with confirmed evidence of single- or multi-vessel CAD as defined above
  - History of ischemic or haemorrhagic stroke (>2 months prior to informed consent)
  - Presence of peripheral artery disease (symptomatic or asymptomatic ) documented by one of the following:
    - Previous limb angioplasty, stenting, or bypass surgery
    - Previous limb or foot amputation due to circulatory insufficiency; or angiographic evidence of significant (>50%) peripheral artery stenosis in at least one limb
    - Evidence from a non-invasive measurement of significant (>50% or as reported as hemodynamically significant) peripheral artery stenosis in at least one limb
    - Ankle brachial index of <0.9 in at least one limb
- Signed and dated written informed consent prior to screening in accordance with Good Clinical Practice and local legislation

**Exclusion criteria**

- Uncontrolled hyperglycemia with glucose >240 mg/dL (>13.3 mmol/L) after an overnight fast during placebo run-in and confirmed by a second measurement (not on the same day)
- Indication of liver disease, defined by serum levels of alanine amininotransferase, aspartate aminotransferase, or alkaline phosphatase above 3 x upper limit of normal (ULN) during screening or run-in phase
- Planned cardiac surgery or angioplasty within 3 months
- Estimated glomerular filtration rate <30 ml/min/1.73m^2^ (according to the Modification of Diet in Renal Disease equation) at screening or during run-in phase
- Bariatric surgery within the past two years and other gastrointestinal surgeries that induce chronic malabsorption
- Blood dyscrasias or any disorders causing hemolysis or unstable red blood cells e.g. malaria, babesiosis, hemolytic anemia)
- Medical history of cancer (except for basal cell carcinoma) and/or treatment for cancer within the last 5 years
- Contraindications to background therapy according to the local label
- Treatment with anti-obesity drugs 3 months prior to informed consent or any other treatment at time of screening leading to unstable body weight (e.g. surgery, aggressive diet regimen, etc.)
- Treatment with systemic steroids at time of informed consent or change in dosage of thyroid hormones within 6 weeks prior to informed consent
- Any uncontrolled endocrine disorder except T2DM
- Pre-menopausal women (last menstruation ≤1 year prior to informed consent) who were nursing, pregnant, or of child-bearing potential and were not practicing an acceptable method of birth control, or did not plan to continue using this method throughout the study, or did not agree to submit to periodic pregnancy testing during the trial
  - Acceptable methods of birth control include tubal ligation, transdermal patch, intrauterine devices/systems, oral, implantable or injectable contraceptives, sexual abstinence, double barrier method, vasectomy of partner
- Alcohol or drug abuse within 3 months of informed consent that would interfere with trial participation or any ongoing condition leading to decreased compliance with study procedures or study drug intake
- Intake of an investigational drug in another trial within 30 days prior to intake of study medication in this trial or participating in another trial involving an investigational drug and/or follow-up
- Any clinical condition that would jeopardize patient safety while participating in this clinical trial (in Canada, this included current genito-urinal infection or genito-urinal infection within 2 weeks prior to informed consent)
- Acute coronary syndrome, stroke, or transient ischemic attack within 2 months prior to informed consent
- In South Africa: blood pressure >160/100 mmHg at screening
