# Supplementary material for: Rationale, design, and baseline characteristics of a randomized, placebo-controlled cardiovascular outcome trial of empagliflozin (EMPA-REG OUTCOME™)
Source: Cardiovasc Diabetol. 2014 Jun 19;13:102. doi: 10.1186/1475-2840-13-102 (PMC4072621; doi:10.1186/1475-2840-13-102)
Supplement: Additional file 2 — Criteria for the institution of rescue therapy. [file 1475-2840-13-102-S2.docx]

**Additional file 2. Criteria for the institution of rescue therapy**

- Background glucose-lowering therapy was to remain unchanged for the first 12 weeks after randomization if possible, although rescue therapy could be initiated. After this period, therapy was allowed adjusted to achieve desired glycemic control at the investigator’s discretion to achieve best standard of care according to local guidelines

**First 12 weeks of treatment**

- For the first 12 weeks after randomization, rescue medication for the treatment of hyperglycemia was allowed to be initiated if the patient had a glucose level >240 mg/dL (>13.3 mmol/L) after an overnight fast (glucose level >200 mg/dL [>11.1 mmol/l] in France), confirmed by a second measurement
  - At least one measurement should be performed after an overnight fast at the investigational site, and on a different day to the initial (overnight fast) measurement
- The initiation, choice, and dosage of rescue medication is at the investigator’s discretion
  - Rescue medication can include uptitration of background therapy
  - If insulin is part of the background therapy, changes of >10% of the total daily prescribed dose of insulin are considered rescue therapy
  - Other SGLT2 inhibitors (if available) were not permitted as rescue medication (neither was pioglitazone in Japan)
- All rescue medication should be taken in accordance with the local prescribing information, considering potential contraindications
- In the case of symptomatic hypoglycemia or severe hypoglycemia, appropriate adjustment of antidiabetes therapy, such as a dose reduction or discontinuation of rescue medication or background therapy, can be initiated
  - Reduction or discontinuation of rescue medication should be considered before reduction in background therapy

**After week 12**

- For participants in Portugal, rescue therapy, as described above, was also allowed from week 12 of treatment onwards:
  - Week 12–28 of treatment if glucose level >200 mg/dL (>11.1 mmol/L) after an overnight fast
  - Week 28 to the end of the trial if glucose level >180 mg/dL (>10.0 mmol/L) and/or HbA_1c_ >8.0% (>64 mmol/mol) after an overnight fast
- After week 12, investigators were permitted to add anti-diabetes treatment to achieve the best standard of care according to local guidelines, based on FPG and HbA_1c_
