# Supplementary material for: Rationale, design, and baseline characteristics of a randomized, placebo-controlled cardiovascular outcome trial of empagliflozin (EMPA-REG OUTCOME™)
Source: Cardiovasc Diabetol. 2014 Jun 19;13:102. doi: 10.1186/1475-2840-13-102 (PMC4072621; doi:10.1186/1475-2840-13-102)
Supplement: Additional file 3 — Outcome definitions for major clinical outcomes. [file 1475-2840-13-102-S3.docx]

**Additional file 3. Outcome definitions for major clinical outcomes**

Cardiovascular death

The cause of death will be determined by the principal condition that caused the death, not the immediate mode of death. Clinical Events Committee (CEC) members will review all available information and use their clinical expertise to adjudicate the cause of death. All deaths not attributed to the categories of CV death and not attributed to a non-CV cause are presumed CV deaths and are part of the CV mortality outcome. Death certificates or summary, if possible, will be provided for all patients who have died, including date and details surrounding death. However, if a death certificate is the only information available for review besides the patient profile in the clinical trial database, the CEC may decide not to use this information as cause of death if another etiology appears more plausible. The following definitions will be used for the adjudication of fatal cases:

**Sudden cardiac death**. Death that occurs unexpectedly in a previously stable patient and includes the following deaths:

- Witnessed and instantaneous without new or worsening symptoms
- Witnessed within 60 minutes of the onset of new or worsening cardiac symptoms
- Witnessed and attributed to an identified arrhythmia (e.g., captured on ECG recording or witnessed on a monitor by either a medic or paramedic)
- Subjects unsuccessfully resuscitated from cardiac arrest or successfully resuscitated from cardiac arrest but who die within 24 hours without identification of a non-cardiac etiology
- Un-witnessed death and there is no conclusive evidence of another, non-CV, cause of death (i.e. presumed CV death)

**Sudden death due to acute MI (MI type 3).** Sudden death occurring up to 14 days after a documented acute MI (verified either by the diagnostic criteria outlined for acute MI or by autopsy findings showing recent MI or recent coronary thrombus) and where there is no conclusive evidence of another cause of death. If death occurs before biochemical confirmation of myocardial necrosis can be obtained, adjudication should be based on clinical presentation and ECG evidence.

**Death due to heart failure or cardiogenic shock.** Death occurring in the context of clinically worsening symptoms and/or signs of congestive heart failure (CHF) without evidence of another cause of death.

New or worsening signs and/or symptoms of CHF include any of the following:

- New or increasing symptoms and/or signs of heart failure requiring the initiation of, or an increase in, treatment directed at heart failure or occurring in a patient already receiving maximal therapy for heart failure
- Heart failure symptoms or signs requiring continuous intravenous therapy or oxygen administration
- Confinement to bed predominantly due to heart failure symptoms
- Pulmonary edema sufficient to cause tachypnea and distress not occurring in the context of an acute myocardial infarction or as the consequence of an arrhythmia occurring in the absence of worsening heart failure
- Cardiogenic shock not occurring in the context of an acute MI or as the consequence of an arrhythmia occurring in the absence of worsening heart failure
  - Cardiogenic shock is defined as SBP <90 mmHg for more than 1 hour, not responsive to fluid resuscitation and/or heart rate correction, and felt to be secondary to cardiac dysfunction and associated with at least one of the following signs of hypoperfusion:
    - Cool, clammy skin
    - Oliguria (urine output < 30 mL/hour)
    - Altered sensorium
    - Cardiac index < 2.2 L/min/m^2^
  - Cardiogenic shock can also be defined in the presence of SBP ≥90 mmHg or for a time period <1 hour if the blood pressure measurement or the time period is influenced by the presence of positive inotropic or vasopressor agents alone and/or with mechanical support <1 hour. The outcome of cardiogenic shock will be based on CEC assessment and must occur after randomization. Episodes of cardiogenic shock occurring before and continuing after randomization will not be part of the study outcome. This category will include sudden death occurring during an admission for worsening heart failure

**Death due to stroke, cerebrovascular event.** Death occurring up to 30 days after a stroke that is either due to the stroke or caused by complication of the stroke.

**Death due to other CV causes.** Death must be due to a fully documented CV cause not included in the above categories (e.g. dysrythmia, pulmonary embolism, or CV intervention). Death due to a MI that occurs as a direct consequence of a CV investigation/procedure/ operation will be classified as death due to other CV cause.

Non-CV death

Non-CV death is defined as any death not covered by cardiac death or vascular death. The CEC will be asked to indicate the most likely cause of non-CV death. Examples of non-CV death are: pulmonary causes, renal causes, gastrointestinal causes, infection (including sepsis), non-infectious (e.g., systemic inflammatory response syndrome (SIRS)), malignancy (i.e., new malignancy, worsening of prior malignancy), hemorrhage (not intracranial), accidental/trauma, suicide, non-CV organ failure (e.g., hepatic failure) or non-CV surgery.

MI (non-fatal)

The term MI should be used when there is evidence of myocardial necrosis in a clinical setting consistent with myocardial ischemia. Under these conditions, any one of the following criteria A to C meets the diagnosis for myocardial infarction.

A. Spontaneous MI (type 1)

To identify a type 1 MI, patients should demonstrate spontaneous symptoms of myocardial ischemia unprovoked by supply/demand inequity, together with ≥1 of the following criteria:

- Cardiac biomarker elevation: Troponin is the preferred marker for use to adjudicate the presence of acute MIfarction. At least one value should show a rise and/or fall above the lowest cut-point providing 10% imprecision (typically the upper reference limit for the troponin run per standard of clinical care). Creatine kinase-MB is a secondary choice to troponin; a rise of CK-MB above the local upper reference limit would be consistent with myocardial injury
- ECG changes consistent with new ischemic changes
  - ECG changes indicative of new ischemia (new ST-T changes or new left bundle branch block [LBBB]) or ECG manifestations of acute myocardial ischemia (in absence of left ventricular hypertrophy [LVH] and LBBB):
  - Development of pathological Q waves in the ECG
    - Any Q-wave in leads V2-V3 ≥0.02 seconds or QS complex in leads V2 and V3
    - Q-wave ≥ 0.03 seconds and ≥0.1 mV deep or QS complex in leads I, II, aVL, aVF, or V4-V6 in any two leads of a contiguous lead grouping (I, aVL, V6; V4-V6; II, III, and aVF)
  - ST elevation: New ST elevation at the J-point in two contiguous leads with the cut-off points: ≥0.2 mV in men or ≥ 0.15 mV in women in leads V2-V3 and/or ≥0.1 mV in other leads
  - ST depression and T-wave changes: New horizontal or down-sloping ST depression ≥0.05 mV in two contiguous leads; and/or T inversion ≥0.1 mV in two contiguous leads with prominent R-wave or R/S ratio >1
- Imaging evidence of new non-viable myocardium or new wall motion abnormality

B. “Demand” related (type 2) MI

Patients with type 2 MI should be considered with similar diagnostic criteria as a type 1 MI, however type 2 MI should be considered present when myocardial ischemia and infarction are consequent to supply/demand inequity, rather than a spontaneous plaque rupture and coronary thrombosis.

C. Percutaneous Coronary Intervention (PCI)-related MI (type 4a/4b)

For PCI in patients with normal baseline troponin values, elevations of cardiac biomarkers above the 99th percentile URL within 24 hours of the procedure are indicative of peri-procedural myocardial necrosis. By convention, increases of biomarkers >3 x 99th percentile URL (troponin or CK-MB >3 x 99th percentile URL) are consistent with PCI-related MI.

If the cardiac biomarker is elevated prior to PCI, a ≥20% increase of the value in the second cardiac biomarker sample within 24 hours of PCI and documentation that cardiac biomarker values were decreasing (two samples ≥6 hours apart) prior to the suspected recurrent MI is consistent with PCI-related MI.

Symptoms of cardiac ischemia are not required.

D. Coronary Artery Bypass Grafting (CABG)-related MI (type 5)

For CABG in patients with normal baseline troponin values, elevation of cardiac biomarkers above the 99th percentile URL within 72 hours of the procedure is indicative of peri-procedural myocardial necrosis. By convention, an increase of biomarkers >5 x 99th percentile URL (troponin or CK-MB >5 x 99th percentile URL) plus at least one of the following

- New pathological Q waves in at least 2 contiguous leads on the electrocardiogram that persist through 30 days or new LBBB
- Angiographically documented new graft or native coronary artery occlusion
- Imaging evidence of new loss of viable myocardium is consistent with CABG-related MI

If the cardiac biomarker is elevated prior to CABG, a ≥20% increase of the value in the second cardiac biomarker sample within 72 hours of CABG and documentation that cardiac biomarker values were decreasing (two samples ≥6 hours apart) prior to the suspected recurrent MI plus new pathological Q waves in ≥2 contiguous leads on the electrocardiogram or new LBBB, angiographically documented new graft or native coronary artery occlusion, or imaging evidence of new loss of viable myocardium is consistent with a periprocedural MI after CABG. Symptoms of cardiac ischemia are not required.

**Clinical classification of acute MI.** For every MI identified by the CEC, one of the following will be assigned:

- Type 1: Spontaneous MI related to ischemia due to a primary coronary event such as plaque erosion and/or rupture, fissuring, or dissection
- Type 2: MI secondary to ischemia due to either increased oxygen demand or decreased supply, e.g. coronary artery spasm, coronary embolism, anemia, arrhythmias, hypertension, or hypotension
- Type 3: Sudden unexpected cardiac death, including cardiac arrest, often with symptoms suggestive of myocardial ischemia, accompanied by presumably new ST elevation, or new LBBB, or evidence of fresh thrombus in a coronary artery by angiography and/or at autopsy, but death occurring before blood samples could be obtained, or at a time before the appearance of cardiac biomarkers in the blood
- Type 4a: MI associated with PCI
- Type 4b: MI associated with stent thrombosis as documented by angiography or at autopsy
- Type 5: MI associated with CABG

Hospitalization for unstable angina

The date of this event will be the day of hospitalization of the patient including any overnight stay at an emergency room or chest pain unit. Unstable angina requiring hospitalization is defined as all of the following:

- No elevation in cardiac biomarkers (cardiac biomarkers are negative for myocardial necrosis) according to conventional assays or contemporary sensitive assays
- Clinical presentation: Cardiac symptoms lasting ≥10 minutes and considered to be myocardial ischemia on final diagnosis with one of the following:
  - Rest angina
  - New-onset (<2 months) severe angina (Canadian Cardiovascular Society [CCS] Grading Scale, or CCS classification system, classification severity ≥III)
  - Increasing angina (in intensity, duration, and/or frequency) with an increase in severity of >1 CCS class to CCS class >III
- Requiring an unscheduled visit to a healthcare facility and overnight admission
- At least one of the following:
  - New or worsening ST or T wave changes on ECG. ECG changes should satisfy the following criteria for acute myocardial ischemia in the absence of LVH and LBBB:
    - ST elevation: New transient (known to be <20 minutes) ST elevation at the J-point in two contiguous leads with the cut-off points - ≥0.2 mV in men or ≥0.15 mV in women in leads V2-V3 and/or ≥ 0.1 mV in other leads
    - ST depression and T-wave changes: New horizontal or down-sloping ST depression ≥ 0.05 mV in two contiguous leads; and/or T inversion ≥0.1 mV in two contiguous leads with prominent R-wave or R/S ratio >1
  - Evidence of ischemia on stress testing with cardiac imaging
  - Evidence of ischemia on stress testing with angiographic evidence of ≥70% lesion and/or thrombus in an epicardial coronary artery or initiation/increased dosing of antianginal therapy
  - Angiographic evidence of ≥70% lesion and/or thrombus in an epicardial coronary artery

Stent thrombosis

Timing

| **Class** | **Description of stage** | | |
| --- | --- | --- | --- |
| Class I | “Ordinary physical activity does not cause . . . angina,” such as walking or climbing stairs. Angina occurs with strenuous, rapid, or prolonged exertion at work or recreation | | |
| Class II | “Slight limitation of ordinary activity.” Angina occurs on walking or climbing stairs rapidly; walking uphill; walking or stair climbing after meals; in cold, in wind, or under emotional stress; or only during the few hours after awakening. Angina occurs on walking more than 2 blocks on the level and climbing more than 1 flight of ordinary stairs at a normal pace and under normal conditions | | |
| Class III | “Marked limitations of ordinary physical activity.” Angina occurs on walking 1 to 2 blocks on the level and climbing 1 flight of stairs under normal conditions and at a normal pace | | |
| Class IV | “Inability to carry on any physical activity without discomfort—anginal symptoms may be present at rest” | | |
| **Type** | | | **Timing** |
| Early stent  thrombosis | | Acute stent thrombosis | 0 to 24 hours after stent implantation |
|  |  | Subacute stent thrombosis | >24 hours to 30 days after stent implantation |
| Late stent thrombosis* | | | >30 days to 1 year after stent implantation |
| Very late stent thrombosis* | | | >1 year after stent implantation |

Stent thrombosis should be reported as a cumulative value over time and at the various individual time points specified above. Time 0 is defined as the time point after the guiding catheter has been removed and the patient has left the catheterization laboratory

*Includes primary as well as secondary late stent thrombosis; secondary late stent thrombosis is a stent thrombosis after a target lesion revascularization

**Definitions of definite, probable, and possible stent thrombosis**

Definite stent thrombosis is considered to have occurred by either angiographic or pathological confirmation:

- Angiographic confirmation of stent thrombosis: The presence of an intracoronary thrombus that originates in the stent or in the segment 5 mm proximal or distal to the stent and presence of ≥1 of the following criteria within a 48-hour time window:
  - Acute onset of ischemic symptoms at rest
  - New ischemic ECG changes that suggest acute ischemia
  - Typical rise and fall in cardiac biomarkers (refer to definition of spontaneous MI: troponin or CK-MB >99th percentile of URL, according to conventional assays or contemporary sensitive assays)
  - Nonocclusive thrombus Intracoronary thrombus is defined as a (spheric, ovoid, or irregular) noncalcified filling defect or lucency surrounded by contrast material (on 3 sides or within a coronary stenosis) seen in multiple projections, or persistence of contrast material within the lumen, or a visible embolization of intraluminal material downstream
  - Occlusive thrombus TIMI 0 or TIMI 1 intrastent or proximal to a stent up to the most adjacent proximal side branch or main branch (if originates from the side branch)

NOTE: The incidental angiographic documentation of stent occlusion in the absence of clinical signs or symptoms is not considered a confirmed stent thrombosis (silent occlusion)

- Pathological confirmation of stent thrombosis Evidence of recent thrombus within the stent determined at autopsy or via examination of tissue retrieved following thrombectomy

Probable Stent Thrombosis is considered to have occurred after intracoronary stenting in the following cases:

- Any unexplained death within the first 30 days
  - In ST-elevation MI population, one may consider the exclusion of unexplained death within 30 days as evidence of probable stent thrombosis
- Irrespective of the time after the index procedure, any MI that is related to documented acute ischemia in the territory of the implanted stent without angiographic confirmation of stent thrombosis and in the absence of any other obvious cause

Possible Stent Thrombosis is considered to have occurred with any unexplained death from 30 days after intracoronary stenting until end of trial follow-up.

Heart Failure (HF) requiring hospitalization

The date of this event will be the day of hospitalization of the patient including any overnight stay at an emergency room or chest pain unit. HF requiring hospitalization is defined as an event that meets all of the following criteria:

- Requires hospitalization defined as an admission to an inpatient unit or a visit to an emergency department that results in at least a 12 hour stay (or a date change if the time of admission/discharge is not available)
- Clinical manifestations of heart failure (new or worsening) including at least one of the following:
  - Dyspnea
  - Orthopnea
  - Paroxysmal nocturnal dyspnea
  - Edema
  - Pulmonary basilar crackles
  - Jugular venous distension
  - Third heart sound or gallop rhythm
  - Radiological evidence of worsening heart failure
- Additional/increased therapy: at least one of the following:
  - Initiation of oral diuretic, intravenous diuretic, inotrope, or vasodilator therapy
  - Uptitration of oral diuretic or intravenous therapy, if already on therapy
  - Initiation of mechanical or surgical intervention (mechanical circulatory support, heart transplantation or ventricular pacing to improve cardiac function), or the use of ultrafiltration, hemofiltration, or dialysis that is specifically directed at treatment of heart failure

Changes in biomarker (e.g., brain natriuretic peptide) consistent with CHF will support this diagnosis.

Coronary revascularization procedure

Either CABG or PCI (e.g., angioplasty, coronary stenting).

- CABG: the successful placement of at least one conduit with either a proximal and distal anastomosis or a distal anastomosis only
- PCI: Successful balloon inflation with or without stenting and the achievement of a residual stenosis <50%. The balloon inflation and/or stenting could have been preceded by device activation (e.g., angiojet, directional coronary atherectomy, or rotational atherectomy)

In cases where the procedure leads to a MI (type 4a, 4b or 5) the event will be adjudicated as an MI.

Transient Ischemic Attack (TIA)

TIA: a transient episode of neurological dysfunction caused by focal brain, spinal cord, or retinal ischemia, without acute infarction.

Stroke

Stroke: the rapid onset of a new persistent neurologic deficit attributed to an obstruction in cerebral blood flow and/or cerebral hemorrhage with no apparent non-vascular cause (e.g., trauma, tumor, or infection). Available neuroimaging studies will be considered to support the clinical impression and to determine if there is a demonstrable lesion compatible with an acute stroke. Strokes will be classified as ischemic, hemorrhagic, or unknown.

**Diagnosis of stroke**. For the diagnosis of stroke, the following 4 criteria should be fulfilled:

- Rapid onset of a focal/global neurological deficit with at least one of the following:
  - Change in level of consciousness
  - Hemiplegia
  - Hemiparesis
  - Numbness or sensory loss affecting one side of the body
  - Dysphasia/aphasia
  - Hemianopia (loss of half of the field of vision of one or both eyes)
  - Other new neurological sign(s)/symptom(s) consistent with stroke

NOTE: If the mode of onset is uncertain, a diagnosis of stroke may be made provided that there is no plausible non-stroke cause for the clinical presentation

- Duration of a focal/global neurological deficit ≥24 hours OR < 24 hours if this is because of at least one of the following therapeutic interventions:
  - Pharmacologic (i.e., thrombolytic drug administration)
  - Non-pharmacologic (i.e., neurointerventional procedure [e.g. intracranial angioplasty])

OR

- - Available brain imaging clearly documents a new hemorrhage or infarct

OR

- - The neurological deficit results in death
- No other readily identifiable non-stroke cause for the clinical presentation (e.g., brain tumor, trauma, infection, hypoglycemia, peripheral lesion)
- Confirmation of the diagnosis by at least one of the following:*
  - Neurology or neurosurgical specialist
  - Brain imaging procedure (at least one of the following):
    - CT scan
    - MRI scan
    - Cerebral vessel angiography
  - Lumbar puncture (i.e. spinal fluid analysis diagnostic of intracranial hemorrhage)

If a stroke is reported but evidence of confirmation of the diagnosis by the methods outlined above is absent, the event will be discussed at a full CEC meeting. In such cases, the event may be adjudicated as a stroke on the basis of the clinical presentation alone, but full CEC consensus will be mandatory.

If the acute focal signs represent a worsening of a previous deficit, these signs must have either

- Persisted for more than one week

OR

- Persisted for more than 24 hours and were accompanied by an appropriate new CT or MRI finding

**Classification of stroke.** Strokes are sub-classified as follows:

- Ischemic (Non-hemorrhagic): A stroke caused by an arterial obstruction due to either a thrombotic (e.g., large vessel disease/atherosclerotic or small vessel disease/lacunar) or embolic etiology. This category includes ischemic strokes with hemorrhagic transformation (i.e. no evidence of hemorrhage on an initial imaging study but appearance on a subsequent scan)
- Hemorrhagic: A stroke due to a hemorrhage in the brain as documented by neuroimaging or autopsy. This category will include strokes due to primary intracerebral hemorrhage (intraparenchymal or intraventricular), subdural hematoma and primary subarachnoid hemorrhage
- Not assessable: The stroke type could not be determined by imaging or other means (e.g., lumbar puncture, neurosurgery, or autopsy) or no imaging was performed.
