# Supplementary material for: Rationale, design, and baseline characteristics of a randomized, placebo-controlled cardiovascular outcome trial of empagliflozin (EMPA-REG OUTCOME™)
Source: Cardiovasc Diabetol. 2014 Jun 19;13:102. doi: 10.1186/1475-2840-13-102 (PMC4072621; doi:10.1186/1475-2840-13-102)
Supplement: Additional file 4 — Study outcomes (non-exhaustive list). [file 1475-2840-13-102-S4.docx]

**Additional file 4. Study outcomes (non-exhaustive list)**

Cardiovascular (CV) outcomes

- Primary outcome
  - Time to the first occurrence of any of the following adjudicated components of the primary composite outcome (3P-MACE): CV death (including fatal stroke and fatal MI), non-fatal MI (excluding silent MI), and non-fatal stroke
- Key secondary CV outcome
  - Time to the first occurrence of any of the following adjudicated components of the secondary composite outcome (4P-MACE): CV death (including fatal stroke and fatal MI), non-fatal MI (excluding silent MI), non-fatal stroke, hospitalization for unstable angina pectoris
- Secondary CV outcome: the occurrence of, and time to, each of the following events:
  - Silent MI
  - Heart failure requiring hospitalization
  - New onset albuminuria (albumin:creatinine ratio [ACR] ≥30 mg/g)
  - New onset macroalbuminuria (ACR ≥300 mg/g)
  - Composite microvascular outcome (and individual components) defined as:

1) Initiation of retinal photocoagulation

2) Vitreous hemorrhage

3) Diabetes-related blindness

4) New or worsening nephropathy defined as:

4a) New-onset macroalbuminuria

4b) Doubling of serum creatinine level accompanied by an eGFR (based on MDRD formula) ≤45 mL/min/1.73m^2^

4c) Initiation of continuous renal replacement therapy

4d) Death due to renal disease

- Exploratory CV outcomes: including, but not limited to the occurrence of, and time to, each of the following adjudicated events:
  - CV death (including fatal stroke and fatal MI)
  - Non-fatal MI
  - Non-fatal stroke
  - Hospitalization for unstable angina
  - All-cause mortality
  - Transient ischemic attack (TIA)
  - Coronary revascularization procedures

Further safety outcomes: including, but not limited to:

- Changes from baseline in ECG, physical examination, vital signs (blood pressure and pulse rate), and laboratory parameters
- Adverse events (AEs, between start of treatment and end of treatment plus 7 days), and AEs of special interest
- Hypoglycemic events (between start of treatment and end of treatment plus 7 days)
- Time to (first) use of rescue medication

Further efficacy (diabetes-related) outcomes: including, but not limited to:

- Change from baseline in HbA_1c_, FPG, weight, waist circumference, and blood pressure at 12 weeks, 52 weeks, once a year until the end of the study, and at study end (and at follow-up for weight and waist circumference)
- Composite outcome of the following conditions at each of 12, 52 weeks, once a year and end of study (all three components of the composite fulfilled)
  - HbA_1c_ reduction ≥0.5%
  - Reduction in systolic blood pressure (SBP) >3 mmHg
  - Reduction in body weight >2%
