# Supplementary material for: Rationale, design, and baseline characteristics of a randomized, placebo-controlled cardiovascular outcome trial of empagliflozin (EMPA-REG OUTCOME™)
Source: Cardiovasc Diabetol. 2014 Jun 19;13:102. doi: 10.1186/1475-2840-13-102 (PMC4072621; doi:10.1186/1475-2840-13-102)
Supplement: Additional file 5 — Study organization. [file 1475-2840-13-102-S5.docx]

**Additional file 5. Study organization**

Steering Committee

Bernard Zinman (Chair), Mount Sinai Hospital, Toronto, ON, Canada; Roberto Ferrari, S. Anna University Hospital, Ferrara, Italy; David Fitchett, St Michael’s Hospital, Toronto, ON, Canada; Silvio Inzucchi, Yale University School of Medicine, New Haven, CT; John Lachin, The George Washington University, Rockville, MD; Christopher Wanner, Universitätsklinik Würzburg, Würzburg, Germany

Voting representatives from sponsor: Uli C. Broedl, Boehringer Ingelheim Pharma GmbH & Co. KG, Ingelheim, Germany; Odd Erik Johansen, Boehringer Ingelheim Norway KS, Asker, Norway; Hans-Jürgen Woerle, Boehringer Ingelheim Pharma GmbH & Co. KG, Ingelheim, Germany

Non-voting representatives from sponsor: Erich Bluhmki, Boehringer Ingelheim Pharma GmbH & Co. KG, Ingelheim, Germany; Jennifer Newman, Boehringer Ingelheim Pharmaceuticals, Inc., Ridgefield, CT

Data Monitoring Committee

Francine K. Welty (Chair), Beth Israel Deaconess Medical Center, Boston, USA; Klaus G. Parhofer, University of Munich, Munich, Germany; Terje R. Pedersen, Oslo University Hospital, Oslo, Norway; Tim Clayton, London School of Hygiene and Tropical Medicine, London, UK; Stuart Pocock (Ad Hoc), London School of Hygiene and Tropical Medicine, London, UK; Mike Palmer (Independent Statistician), N Zero 1 Ltd, Wilmslow, UK

Clinical Event Committee Cardiology Peter Clemmensen; Peer Grande; Steen Pehrson: all Heart Center of ‘Rigshospitalet’,the Copenhagen University Hospital, Copenhagen, Denmark. James Januzzi Jr ; Malissa J. Wood: both Massachusetts General Hospital, Boston, USA; Mark Petrie, Golden Jubilee National Hospital, Glasgow, Scotland

Clinical event committee Neurology Tiina Sairanen, Turgut Tatlisumak, Lauri Soinne; all Helsinki University Central Hospital, Helsinki, Finland; Carlos Kase, Dept. of Neurology, Boston University Medical Center, Boston, USA; Tanya Turan; MUSC Stroke Program, Dept. of Neurosciences, Charleston, USA
