# Supplementary material for: Rationale, design, and baseline characteristics of a randomized, placebo-controlled cardiovascular outcome trial of empagliflozin (EMPA-REG OUTCOME™)
Source: Cardiovasc Diabetol. 2014 Jun 19;13:102. doi: 10.1186/1475-2840-13-102 (PMC4072621; doi:10.1186/1475-2840-13-102)
Supplement: Additional file 6 — Selected subgroups of interest. [file 1475-2840-13-102-S6.docx]

**Additional file 6. Selected subgroups of interest**

Categories of selected covariates for displays of baseline characteristics, subgroup analyses and statistical assessmet of primary and key secondary outcomes

| **Variable at baseline** | **Category of covariate** | | |
| --- | --- | --- | --- |
|  | Version 1 | Version 2 | Version 3 |
| Age (years) | <50  50 to <65  65 to <75  ≥75 | < 65  ≥65 |  |
| CV complications | - Only cerebrovascular disease  - Only cardiac disease  - Only peripheral artery disease  - 2 of the 3 above  - All 3 of the above |  |  |
| HbA1c (%) | <8.5  ≥8.5 | <8  8 to <9  ≥9 | <8.0  ≥8.0 |
| BMI (kg/m2) | <30  ≥30 | <25  25 to <30  30 to <35  ≥35 | <25  ≥25 |
| Weight (kg) | ≤70  >70 to ≤80  >80 to ≤ 90  >90 | ≤50  >50 to ≤70  >70 to ≤ 90  >90 |  |
| Geographical  region | Europe  North America  Latin America  Africa/Middle East  Asia |  |  |
| Race | White  Black/African  Asian  Other |  |  |
| Gender | Male  Female |  |  |
| Ethnicity | Hispanic/Latino  Not Hispanic/Latino |  |  |
| Time since diagnosis of diabetes (years) | <= 1  >1 to 5  >5 to 10  >10 |  |  |
| Renal function (MDRD or CG formulae) | >=90  60 to <90  30 to <60  <30 | >=90  60 to <90  45 to <60  30 to <45  <30 |  |
| Blood pressure | ≥ 130/80mmHg  < 130/80mmHg |  |  |
| Cohort characteristics | Enrolled before amendment Q4 2011  Enrolled after amendment Q4 2011 |  |  |
